# Effects of intraoperative inspired oxygen fraction (FiO_2_ 0.3 vs 0.8) on patients undergoing off-pump coronary artery bypass grafting: the CARROT multicenter, cluster-randomized trial

**DOI:** 10.1186/s13054-023-04558-8

**Published:** 2023-07-13

**Authors:** Karam Nam, Jae-Sik Nam, Hye-Bin Kim, Jaeyeon Chung, In Eob Hwang, Jae-Woo Ju, Jinyoung Bae, Seohee Lee, Youn Joung Cho, Jae-Kwang Shim, Young-Lan Kwak, Ji-Hyun Chin, In-Cheol Choi, for the CARdiac suRgery and Oxygen Therapy (CARROT) Investigators, Eun-Ho Lee, Yunseok Jeon

**Affiliations:** 1 Department of Anesthesiology and Pain Medicine, Seoul National University Hospital, Seoul National University College of Medicine, 101 Daehak-ro, Jongno-gu, Seoul, 03080 Republic of Korea; 2 Department of Anesthesiology and Pain Medicine, Asan Medical Center, University of Ulsan College of Medicine, 88 Olympic-ro 43-gil, Songpa-gu, Seoul, 05505 Republic of Korea; 3 Department of Anesthesiology and Pain Medicine, Severance Hospital, Anesthesia and Pain Research Institute, Yonsei University College of Medicine, Seoul, Republic of Korea; 4 Present Address: Department of Anesthesiology and Pain Medicine, Korea University Guro Hospital, Korea University College of Medicine, Seoul, Republic of Korea; 5 Present Address: Medical Service Corps of the First Logistics Support Command, Wonju, Gangwon State Republic of Korea; 6 Present Address: Department of Anesthesiology and Pain Medicine, Ajou University Medical Center, Ajou University School of Medicine, Suwon, Gyeonggi Province Republic of Korea; 7 Present Address: Hana Anesthesia Clinic, Seoul, Republic of Korea

**Keywords:** Cardiac surgical procedures, Coronary artery bypass, Hyperoxia, Oxygen, Postoperative complications

## Abstract

**Background:**

To maintain adequate oxygenation is of utmost importance in intraoperative care. However, clinical evidence supporting specific oxygen levels in distinct surgical settings is lacking. This study aimed to compare the effects of 30% and 80% oxygen in off-pump coronary artery bypass grafting (OPCAB).

**Methods:**

This multicenter trial was conducted in three tertiary hospitals from August 2019 to August 2021. Patients undergoing OPCAB were cluster-randomized to receive either 30% or 80% oxygen intraoperatively, based on the month when the surgery was performed. The primary endpoint was the length of hospital stay. Intraoperative hemodynamic data were also compared.

**Results:**

A total of 414 patients were cluster-randomized. Length of hospital stay was not different in the 30% oxygen group compared to the 80% oxygen group (median, 7.0 days vs 7.0 days; the sub-distribution hazard ratio, 0.98; 95% confidence interval [CI] 0.83–1.16; *P* = 0.808). The incidence of postoperative acute kidney injury was significantly higher in the 30% oxygen group than in the 80% oxygen group (30.7% vs 19.4%; odds ratio, 1.94; 95% CI 1.18–3.17; *P* = 0.036). Intraoperative time-weighted average mixed venous oxygen saturation was significantly higher in the 80% oxygen group (74% vs 64%; *P* < 0.001). The 80% oxygen group also had a significantly greater intraoperative time-weighted average cerebral regional oxygen saturation than the 30% oxygen group (56% vs 52%; *P* = 0.002).

**Conclusions:**

In patients undergoing OPCAB, intraoperative administration of 80% oxygen did not decrease the length of hospital stay, compared to 30% oxygen, but may reduce postoperative acute kidney injury. Moreover, compared to 30% oxygen, intraoperative use of 80% oxygen improved oxygen delivery in patients undergoing OPCAB.

*Trial registration* ClinicalTrials.gov (NCT03945565; April 8, 2019).

**Supplementary Information:**

The online version contains supplementary material available at 10.1186/s13054-023-04558-8.

## Introduction

Oxygen delivery (DO_2_) to peripheral tissue is frequently threatened during surgery due to various factors such as acute blood loss or large fluid shift [1]. Thus, patients undergoing surgery commonly receive a fraction of inspired oxygen (FiO_2_) higher than that in room air to maintain adequate oxygenation and perfusion [2, 3]. However, this conventional practice may lead to supraphysiological levels of oxygen. The resultant hyperoxia can induce oxidative stress, vasoconstriction, and microcirculatory disturbances [4–7], which may, in turn, exert unfavorable effects on postoperative outcomes. Moreover, it is generally accepted that above a certain level, manipulating FiO_2_ has little effect on DO_2_ [8]. Consequently, using high levels of supplemental oxygen during surgery is controversially discussed [9, 10]. However, evidence regarding the optimal perioperative FiO_2_ is insufficient and inconsistent [11].

Unlike in patients undergoing on-pump coronary artery bypass grafting, hemodynamic instability is very common in patients undergoing off-pump coronary artery bypass grafting (OPCAB) because the beating heart with considerable coronary artery disease is lifted, rotated, and fixated during surgery [12]. Oxygen therapy using a high FiO_2_ has certain advantages in terms of oxygenation and perfusion at the potential expense of complications related to oxygen toxicity. However, no previous study has tested the effects of a high FiO_2_ on clinical outcomes in patients undergoing OPCAB.

The purpose of this study was to assess the effects of intraoperative FiO_2_ on patients undergoing OPCAB. In this multicenter, cluster-randomized trial, we compared clinical outcomes and hemodynamic parameters between patients who received 80% and 30% oxygen during OPCAB.

## Methods

### Study design and outline

The CARdiac suRgery and Oxygen Therapy (CARROT) study is a multicenter, cluster-randomized trial conducted in three tertiary hospitals in Seoul, South Korea, from August 2019 to August 2021. This study was approved by the local ethics committees of the three participating hospitals (the Institutional Review Board of Seoul National University Hospital [SNUH], no. 1902-021-1008; the Institutional Review Board of Asan Medical Center [AMC], no. 2019–0818; and the Severance Hospital Institutional Review Board, no. 4-2020-0741), and the protocol was registered at ClinicalTrials.gov (identifier, NCT03945565). All patients enrolled in this study provided written informed consent. The present study was conducted in compliance with the Declaration of Helsinki and adhered to the CONSORT guidelines.

### Participants and randomization

Adult patients (aged ≥ 19 years) scheduled for elective OPCAB were included in this study. Exclusion criteria comprised robot-assisted surgery, minimally invasive direct coronary artery bypass grafting, concomitant major surgery, primary pulmonary morbidity requiring oxygen therapy before surgery, mechanical ventilation (MV) prior to surgery, preoperative mechanical circulatory support (e.g., extracorporeal membrane oxygenation, intra-aortic balloon pump), and refusal to participate.

In this study, month-by-month cluster randomization was performed; participants were assigned to receive either 30% or 80% oxygen (the 30% and 80% oxygen groups, respectively) during OPCAB, based on the month in which the surgery was performed. As FiO_2_ levels have not been universally tested in the field of cardiac surgery and, thus, guidelines did not specify any particular levels of FiO_2_ [11], we hoped this study to be pragmatic and tried to compare low and high levels of FiO_2_ that are used in real-world clinical practice. Based on our clinical experience in the three participating hospitals and previous studies including two landmark trials performed in noncardiac surgical patients [13–15], we decided on 30% and 80% oxygen as the low and high FiO_2_ levels, respectively, for this study. Before the beginning of participant enrollment, an independent research nurse in one hospital (SNUH) performed the randomization procedure with a block size of two or four using a computer program (R version 3.4.3; R Foundation for Statistical Computing, Vienna, Austria). The random allocation table was password protected, and the monthly group allocation was communicated to each participating hospital every month. While the attending anesthetists could not be blinded to the group allocation, statisticians who assessed the collected data, as well as patients, surgeons, intensivists, and ward physicians, remained blinded.

### Study protocol

In all hospitals, anesthesia was induced with intravenous administration of midazolam and sufentanil or remifentanil and maintained using a target-controlled infusion of propofol and remifentanil. The use of 100% oxygen was allowed during preoxygenation at anesthesia induction. After tracheal intubation, MV was initiated with 30% or 80% oxygen as allocated. If oxygen saturation decreased below 93–94% or arterial oxygen partial pressure (PaO_2_) below 60–80 mmHg, an alveolar recruitment maneuver was performed, and then a positive end-expiratory pressure of 5–10 cmH_2_O was applied when necessary. The FiO_2_ was increased if this safety goal was not achieved despite the rescue therapy or whenever deemed fatal hypoxia by the attending anesthetists. The use of 100% oxygen during transfer to the intensive care unit after the end of surgery was allowed.

Complete coronary revascularization was performed off-pump whenever feasible. Otherwise, routine anesthetic/intraoperative and postoperative care procedures in each participating hospital were not regulated by the protocol of this study, except for the above-mentioned intraoperative FiO_2_ setting. The routine perioperative management of OPCAB patients in each hospital is briefed in Additional file 1: Table S1.

### Primary and exploratory secondary clinical outcomes

The primary clinical outcome was hospital length of stay (LOS) after OPCAB. Evidence has been inconsistent and lacking regarding the effects of the FiO_2_ level during OPCAB on postoperative outcomes [11]. We expected that changes of FiO_2_ level may affect various complications differently. For example, if a higher FiO_2_ may decrease surgical site infection [16], and at the same time, increase lung injury [17], the net effect on the composite complication could be negative. Thus, we decided that measures like the frequency of specific or composite complications may not be appropriate to reflect the clinical effects of different FiO_2_ levels. Instead, we considered hospital LOS the most appropriate clinical outcome to evaluate the clinical effects of different oxygen fractions.

The *exploratory* secondary clinical outcomes included length of intensive care unit stay, MV time, prolonged MV, initial postoperative PaO_2_/FiO_2_ ratio (one out of three participating hospitals), in-hospital mortality, 30-day mortality, delirium, stroke, sternal wound infection (identified by the National Healthcare Safety Network surveillance definition of the Centers for Disease Control and Prevention; see Additional file 1: Tables S2–4), acute kidney injury (AKI) developed within seven days after surgery (defined based on the serum creatinine criteria of the Kidney Disease: Improving Global Outcomes [18]), newly initiated renal replacement therapy, new-onset atrial fibrillation, type 5 myocardial infarction (diagnosed based on the Fourth Universal Definition of Myocardial Infarction [19]), conversion to on-pump surgery, and revascularization within 30 days after surgery. Definitions of the secondary clinical outcomes are provided in Additional file 1.

### Biochemical outcome parameters

Biochemical outcome parameters were the maximum concentrations and areas under the curve of high-sensitivity cardiac troponin T (Severance Hospital), troponin I (SNUH and AMC), and creatine kinase MB (all three participating hospitals) measured within 72 h after surgery, and the first postoperative serum lactate concentration (all three participating hospitals). The postoperative protocols for measurements of these outcome parameters are summarized in Additional file 1: Table S1. In one hospital (SNUH), neutrophil gelatinase-associated lipocalin (NGAL) was also measured at the end of surgery.

### Hemodynamic data

Data from intraoperative arterial blood gas analysis and hemodynamic monitoring were gathered as follows. From intraoperative arterial blood gas analysis, hemoglobin concentration, arterial oxygen saturation, and PaO_2_ were collected. Routine timing of intraoperative arterial blood gas analysis in each participating hospital is presented in Additional file 1: Table S1.

Regarding hemodynamic data, cardiac output (CO) and mixed venous oxygen saturation (SvO_2_) were measured via a pulmonary artery catheter (Swan-Ganz CCOmbo V 774HF75; Edwards Lifesciences, Irvine, CA, USA) being connected to a monitoring device (Vigilance II™; Edwards Lifesciences). Cerebral regional oxygen saturation was measured by near-infrared spectroscopy (INVOS™ Cerebral/Somatic Oximeter; Medtronic, Minneapolis, MN, USA). Mean arterial blood pressure data, recorded automatically every five minutes, were also collected.

### Statistical analysis

We assumed a between-group median difference of 25% in hospital LOS as clinically significant. To detect this difference with a two-sided α error of < 0.05 and a *β* error of < 0.2 while assuming a drop-out rate due to in-hospital mortality of 2% [20] and an additional withdrawal rate of 5%, 414 participants (207 in each group) were required for this study, resulting in 385 participants discharged alive.

After checking the normality assumption for the baseline summary statistics, continuous data are presented as mean (standard deviation) or median (interquartile range) where appropriate. Categorical data are expressed as number (proportion). Then, the balance between the study groups were evaluated with the generalized estimating equations procedure accounting for intra-cluster correlation [21].

The primary clinical outcome, hospital LOS after OPCAB, was estimated using the cumulative incidence function plot and compared between the groups using the Fine and Gray model to adjust for the month-by-month clustered data and account for competing risk of in-hospital mortality.

For analyses of the exploratory secondary clinical and biochemical outcomes, the generalized estimating equations procedure was used, accounting for intra-cluster correlation [21]. The intraoperative arterial blood gas analysis data and hemodynamic variables were summarized as their time-weighted average values. To obtain reliable time-weighted average values, patients with less than three measurements were omitted. Then, an exploratory analysis was performed to compare the values between the groups using the generalized estimating equations, taking into account intra-cluster correlation. To account for the issue of multiple testing, the false discovery rate method was employed to adjust *P* values for all exploratory secondary outcomes.

Data were analyzed using SAS (version 9.4; SAS Institute, Cary, NC, USA) or R (version 4.1.2; R Foundation for Statistical Computing, Vienna, Austria). A two-tailed *P* value < 0.05 was considered statistically significant. The results of all outcome analyses in this study were reported based on the intention-to-treat principle. Per-protocol analyses were performed separately as well.

## Results

**Fig. 1:**
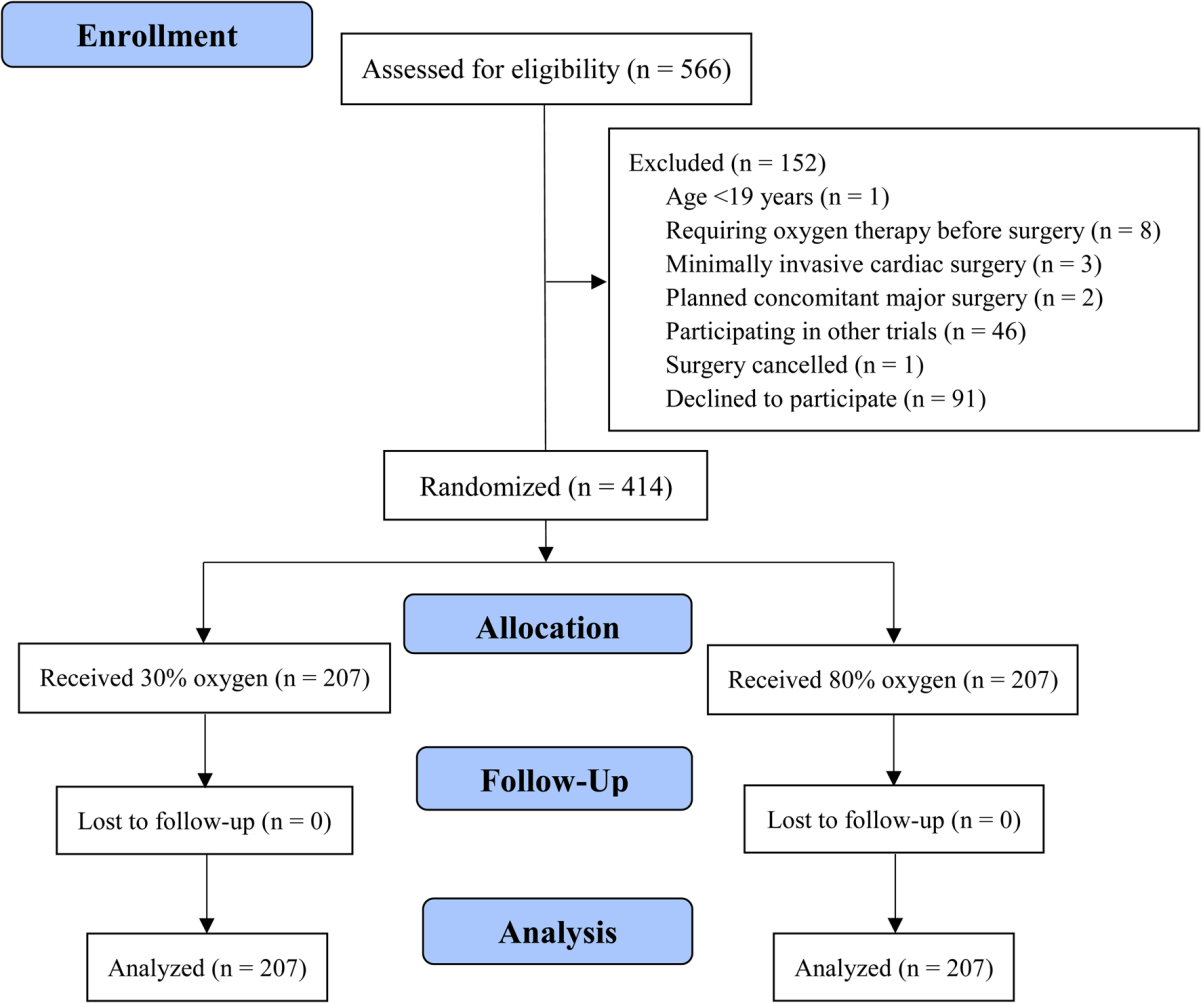
CONSORT flow diagram of the CARROT trial

From August 2019 to August 2021, 566 patients undergoing OPCAB were screened for eligibility (Fig. 1). After excluding 152 patients, 414 patients were cluster-randomized into the 30% or 80% oxygen group (207 participants in each group; an intention-to-treat cohort). The distribution of sample size according to clusters and participating hospitals is shown in Additional file 1: Figure S1. All participants had the primary clinical outcome data available for the planned analysis without loss to follow-up. Baseline characteristics and operative profiles of the participants are provided in Table 1.

**Table 1:** Baseline characteristics and operative profiles

	30% oxygen (*n* = 207)	80% oxygen (*n* = 207)	SMD	*P*
Age, years	65.4 (9.6)	66.2 (8.7)	− 0.089	0.332
Male sex	169 (81.6%)	166 (80.2%)	0.037	0.729
Body mass index, kg/m^2^	24.8 (3.5)	24.9 (3.1)	− 0.034	0.682
Current smoker	34 (16.4%)	26 (12.6%)	0.110	0.276
Hypertension	147 (71.0%)	151 (73.0%)	− 0.043	0.759
Diabetes	123 (59.4%)	120 (58.0%)	0.029	0.763
Dyslipidemia	111 (53.6%)	106 (51.2%)	0.048	0.453
Atrial fibrillation	7 (3.4%)	10 (4.8%)	− 0.073	0.456
Cerebrovascular disease	36 (17.4%)	24 (11.6%)	0.165	0.100
Chronic obstructive pulmonary disease	12 (5.8%)	6 (2.9%)	0.142	0.152
Chronic kidney disease	39 (18.8%)	45 (21.7%)	− 0.072	0.433
ESRD or preoperative RRT	18 (8.7%)	16 (7.7%)	0.035	0.612
Serum creatinine, mg/dl	1.0 (0.8–1.2)	0.9 (0.8–1.1)	0.111	0.181
Hemoglobin, g/dl	12.5 (1.8)	12.7 (1.8)	− 0.104	0.252
Medication history				
Aspirin	191 (92.3%)	195 (94.2%)	− 0.077	0.459
Beta blocker	120 (58.0%)	127 (61.4%)	− 0.069	0.523
Calcium channel blocker	69 (33.3%)	75 (36.2%)	− 0.061	0.526
Statin	171 (82.6%)	174 (84.1%)	− 0.039	0.837
Insulin	29 (14.0%)	27 (13.0%)	0.028	0.712
NYHA classification			0.162	0.262
I	98 (47.3%)	114 (55.1%)		
II	89 (43.0%)	75 (36.2%)		
III	19 (9.2%)	16 (7.7%)		
IV	1 (0.5%)	2 (1.0%)		
Left ventricular ejection fraction, %	55 (12)	56 (11)	− 0.096	0.320
EuroSCORE II, %	1.3 (0.8–2.1)	1.2 (0.8–2.0)	0.072	0.463
Prior myocardial infarction	49 (23.7%)	49 (23.7%)	0.070	0.481
Prior PCI	51 (24.6%)	51 (24.6%)	< 0.001	0.790
Prior CABG	6 (2.9%)	2 (1.0%)	0.141	0.159
Diagnosis of coronary artery disease			0.248	0.687
Stable angina	103 (49.8%)	100 (48.3%)		
Unstable angina	55 (26.6%)	68 (32.9%)		
Acute myocardial infarction	11 (5.3%)	17 (8.2%)		
Others	38 (18.4%)	22 (10.6%)		
No. of diseased coronary arteries			0.205	0.047
1-vessel disease	3 (1.5%)	1 (0.5%)		
2-vessel disease	36 (17.4%)	25 (12.1%)		
3-vessel disease	168 (81.2%)	181 (87.4%)		
Left main coronary artery disease	72 (34.8%)	67 (32.4%)	0.051	0.594
Operative profiles				
Duration of surgery, min	265 (225–304)	260 (230–298)	0.058	0.794
Infused crystalloid, ml	2500 (1750–3275)	2300 (1700–3000)	0.127	0.646
Infused colloid, ml	100 (0–500)	100 (0–500)	0.079	0.826
Transfused packed RBCs, units	0 (0–2)	0 (0–1)	0.311	0.027
Urine output, ml/kg/h	1.3 (0.7–2.3)	1.3 (0.7–2.2)	− 0.020	0.915
Intra-aortic balloon pump use	2 (1.0%)	0 (0.0%)	0.140	NA
No. of distal coronary anastomoses	3 (2–4)	3 (3–4)	− 0.232	0.038
Conduits used for CABG				
Left internal thoracic artery	187 (90.3%)	198 (95.7%)	− 0.209	0.083
Right internal thoracic artery	19 (9.2%)	28 (13.5%)	− 0.137	0.830
Right gastroepiploic artery	1 (0.5%)	1 (0.5%)	< 0.001	0.994
Radial artery	3 (1.4%)	3 (1.4%)	< 0.001	0.991
Saphenous vein	187 (90.3%)	180 (87.0%)	0.107	0.876

Data are expressed as mean (standard deviation), median (interquartile range), or number (proportion)

*SMD* standardized mean difference; *ESRD* End-stage renal disease; *RRT* Renal replacement therapy; *NYHA* Ney York Heart Association; *EuroSCORE* European System for Cardiac Operative Risk Evaluation; *PCI* Percutaneous coronary intervention; *CABG* Coronary artery bypass grafting; *PaO*_*2*_ Arterial oxygen partial pressure; *FiO*_*2*_ Inspired oxygen fraction; *RBC* Red blood cell; *NA* Not applicable

Treatment separation was well achieved between the study groups during OPCAB: the mean time-weighted average PaO_2_ in the 30% and 80% oxygen groups was 124 (33) mmHg and 316 (63) mmHg, respectively. The mean time-weighted average arterial oxygen saturation was 97.5% (1.5) and 99.8% (0.5), respectively. Six patients required the aforementioned rescue therapy (5 out of 207 [2.4%] in the 30% oxygen group and 1 out of 207 [0.5%] in the 80% oxygen group). Sustained hypoxia that did not improve with rescue therapy was not reported in either group. Intraoperative mechanical ventilatory profiles are shown in Additional file 1: Table S5. Nine patients underwent on-pump conversion; the remaining 405 patients were analyzed as a per-protocol cohort.

### Primary and exploratory secondary clinical outcomes

**Fig. 2:**
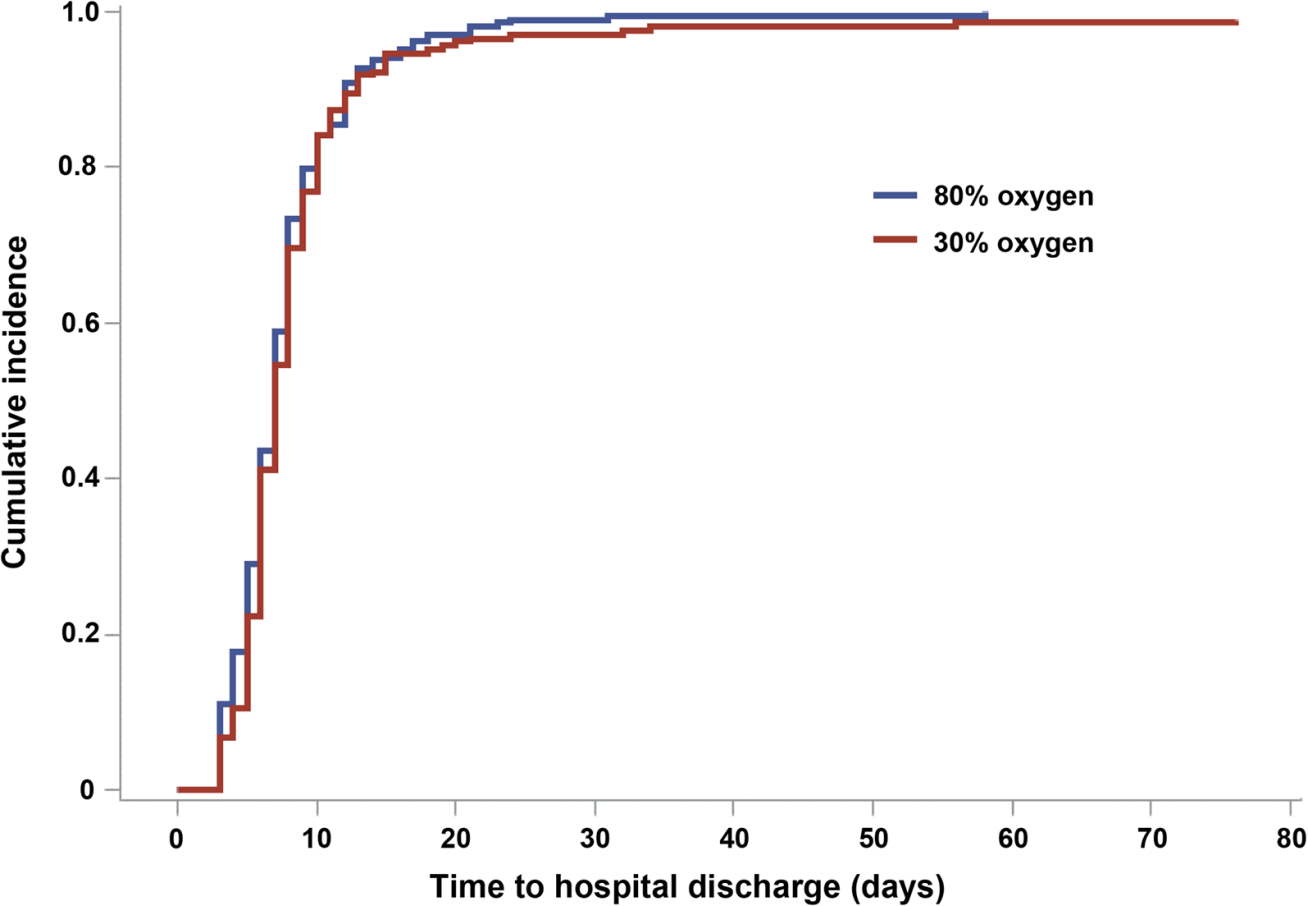
Cumulative incidence function plot of Postoperative hospital length of stay in the 30% versus 80% oxygen groups. *ITT* Intention-to-treat; *sHR* Sub-distribution hazard ratio; *CI* Confidence interval; *PP* Per-protocol

The median (95% confidence interval [CI]) hospital LOS after OPCAB was 7.0 (6.5–7.5) days for the 30% oxygen group and 7.0 (6.6–7.4) days for the 80% oxygen group. There was no significant difference in hospital LOS between the two groups, in both intention-to-treat (the sub-distribution hazard ratio, 0.98; 95% CI 0.83–1.16; *P* = 0.808) and per-protocol analyses (the sub-distribution hazard ratio, 0.95, 95% CI 0.78–1.16, *P* = 0.644; Fig. 2). Data on readmission after discharge from the index hospitalization are summarized in Additional file 1: Table S6.

**Fig. 3:**
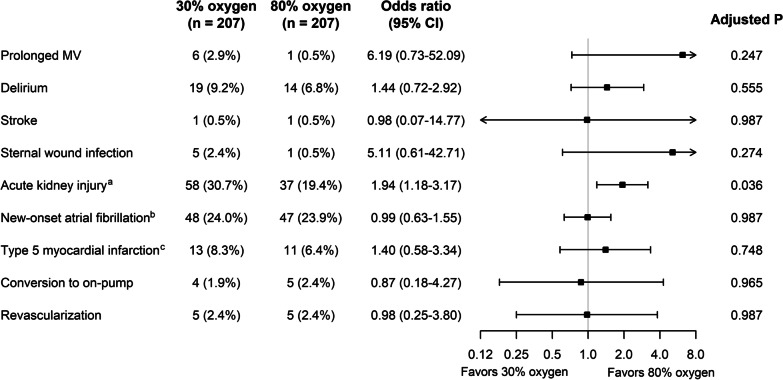
Intention-to-treat analysis of the binary secondary clinical outcomes. CI, confidence interval; MV, mechanical ventilation. ^a^18 and 16 patients with preoperative end-stage renal disease or renal replacement therapy in the 30% and 80% oxygen groups, respectively, were excluded. ^b^7 and 10 patients with a preoperative history of atrial fibrillation in the 30% and 80% oxygen groups, respectively, were excluded. ^c^Missing data in 47 and 31 patients in the 30% and 80% oxygen groups, respectively

There was no significant difference in the exploratory secondary clinical outcomes between both groups, except for AKI (Table 2 and Fig. 3). After excluding 34 patients with preoperative end-stage renal disease or renal replacement therapy, 25.0% (95/380) of the overall intention-to-treat cohort developed AKI after OPCAB. The incidence of AKI was significantly greater in the 30% oxygen group (58/189; 30.7%) than in the 80% oxygen group (37/191; 19.4%; odds ratio, 1.94; 95% CI 1.18–3.17; adjusted *P* = 0.036). Only one patient in the 80% oxygen group required postoperative renal replacement therapy. Two patients in the 30% oxygen group died in hospital because of aspiration, and no additional mortality was reported within 30 days after surgery. There were two cases (one case in each group) of postoperative stroke. Similar results were found in per-protocol analyses (see Additional file 1: Table S7 and Figure S2).

**Table 2:** Intention-to-treat analysis of the continuous secondary clinical outcomes

	30% oxygen (*n* = 207)	80% oxygen (*n* = 207)	Estimate (95% CI)^a^	*P*^b^
Initial postoperative PaO_2_/FiO_2_ ratio^c^	318 (109)	293 (114)	24 (− 5–54)	0.252
ICU length of stay, hours	51.1 (43.6)	53.5 (43.7)	− 2.4 (− 10.8 to 5.7)	0.644
MV time, hours	15.6 (20.2)	12.1 (7.2)	4.1 (− 0.3 to 7.9)	0.102

Data are presented as mean (standard deviation) or number (proportion). Definitions of the outcomes and the results of per-protocol analyses are provided in Additional file 1

CI, confidence interval; PaO_2_, arterial oxygen partial pressure; FiO_2_, inspired oxygen fraction; ICU, intensive care unit; MV, mechanical ventilation

^a^Referenced to the 80% oxygen group

^b^False discovery rate-corrected values

^c^The results are from one participating hospital (*n* = 113 and 103 in the 30% and 80% oxygen groups, respectively)

### Biochemical outcomes

In one participating hospital (Severance Hospital, *n* = 68), serum cardiac troponin T levels were measured. The maximum values and areas under the curve of troponin T levels within 72 h after surgery were significantly higher in the 30% oxygen group than in the 80% oxygen group. In the other two hospitals (SNUH and AMC, *n* = 346), serum cardiac troponin I levels were measured, but the two groups were not significantly different. Likewise, no difference was found in creatine kinase MB levels among the two study groups (all three participating hospitals, *n* = 414; Table 3).

**Table 3:** Intention-to-treat analysis of biochemical outcomes

	30% oxygen (*n* = 207)	80% oxygen (*n* = 207)	Estimate (95% CI)^a^	*P*^*b*^
72 h maximum cTnT, pg/ml^c^	0.52 (0.88)	0.23 (0.22)	0.29 (0.06 to 0.51)	0.041
72 h AUC cTnT, pg/ml·h^c^	26.2 (44.1)	10.3 (8.6)	15.7 (4.6 to 26.7)	0.026
72 h maximum cTnI, ng/ml^d^	2.67 (5.29)	3.03 (5.59)	− 0.36 (− 1.50 to 0.79)	0.748
72 h AUC cTnI, ng/ml·h^d^	64.6 (142.7)	75.3 (160.9)	− 10.8 (− 40.6 to 19.0)	0.748
72 h maximum CKMB, ng/ml	9.98 (19.6)	8.95 (9.21)	1.08 (− 2.05 to 4.21)	0.748
72 h AUC CKMB, ng/ml·h	262.9 (500.6)	248.7 (278.1)	23.6 (− 57.3 to 104.5)	0.748
First postoperative lactate, mmol/l	1.1 (0.5)	1.0 (0.4)	0.08 (− 0.02 to 0.19)	0.271
NGAL, at the end of surgery, ng/ml^e^	133 (205)	91 (91)	41 (3 to 79)	0.102

Data are presented as mean (standard deviation)

CI, confidence interval; cTnT, cardiac troponin T; AUC, area under the curve; cTnI, cardiac troponin I; CKMB, creatine kinase MB; NGAL, neutrophil gelatinase-associated lipocalin

^a^Referenced to the 80% oxygen group

^b^False discovery rate-corrected values

^c^*n* = 25 and 43 in the 30% and 80% oxygen groups, respectively

^d^*n* = 182 and 164 in the 30% and 80% oxygen groups, respectively

^e^*n* = 101 and 96 in the 30% and 80% oxygen groups, respectively

While the first postoperative serum lactate concentration was also comparable between the two groups (all three participating hospitals, *n* = 414; Table 3 and Additional file 1: Figure S3), serum NGAL concentrations measured at the end of surgery (SNUH, *n* = 197) tended to be higher in the 30% oxygen group than in the 80% oxygen group (133 [205] ng/ml vs 91 [91] ng/ml, respectively; adjusted *P* = 0.102).

The per-protocol analysis of these biomarkers showed similar results (see Additional file 1: Table S8).

### Hemodynamic data

While time-weighted average intraoperative hemoglobin concentration and CO were similar for both groups, arterial oxygen saturation (97.9% [1.5] vs 99.8% [0.5]; adjusted *P* = 0.001) and PaO_2_ (124 [33] mmHg vs 316 [63] mmHg; adjusted *P* = 0.001; Table 4) were significantly lower in the 30% oxygen group than in the 80% oxygen group. There was a statistically significant but minimal difference in mean intraoperative arterial blood pressure between the 30% and 80% oxygen groups (74 [6] mmHg vs 76 [6] mmHg; adjusted *P* = 0.024; Table 4). The 30% oxygen group had a 10-percentage point lower time-weighted average SvO_2_ (63.9% [9.7]) than the 80% oxygen group (73.9% [7.3]; adjusted *P* = 0.001) during surgery. The 30% oxygen group also had a lower increased intraoperative cerebral regional oxygen saturation (51.7% [14.5]) compared to the 80% oxygen group (56.4% [16.1]; adjusted *P* = 0.001). The per-protocol analyses are summarized in Additional file 1: Table S9.

**Table 4:** Intention-to-treat analysis of hemodynamic data

	30% oxygen (*n* = 207)	80% oxygen (*n* = 207)	Estimate (95% CI)^a^	*P*^*b*^
Hemoglobin, g/dl^b^	10.3 (1.2)	10.5 (1.4)	− 0.2 (− 0.4 to 0.1)	0.294
SaO_2_, %^b^	97.9 (1.5)	99.8 (0.5)	− 1.8 (− 2.1 to − 1.6)	0.001
PaO_2_, mmHg^b^	124 (33)	316 (63)	− 195 (− 207 to − 183)	0.001
SvO_2_, %^b^	63.9 (9.7)	73.9 (7.3)	− 8.8 (− 10.4 to − 7.2)	0.001
Cardiac output, l/min^b^	3.6 (0.8)	3.6 (0.9)	0.0 (− 0.2 to 0.3)	0.937
Cardiac index, l/min/m^2b^	2.1 (0.4)	2.1 (0.4)	0.0 (− 0.1 to 0.2)	0.748
Cerebral rSO_2_, %^c^	51.7 (14.5)	56.4 (16.1)	− 5.0 (− 6.6 to − 3.5)	0.001
MBP, mmHg	74 (6)	76 (6)	− 1.4 (− 2.4 to − 0.5)	0.024

All values are time-weighted average intraoperative values. Data are presented as mean (standard deviation)

CI, confidence interval; SaO_2_, arterial oxygen saturation; PaO_2_, arterial oxygen partial pressure; SvO_2_, mixed venous oxygen saturation; rSO_2_, regional oxygen saturation; MBP, mean blood pressure

^a^Referenced to the 80% oxygen group

^b^False discovery rate-corrected values

^c^*n* = 149 and 148 in the 30% and 80% oxygen groups, respectively

^d^The lower of the left and right side values was taken

## Discussion

In our study, there was no difference in hospital LOS after OPCAB between the 30% and 80% oxygen groups. However, compared to the 30% oxygen group, there was nearly a 40% reduction of the postoperative AKI incidence in the 80% oxygen group, with a lower level of serum NGAL. In addition, SvO_2_ and cerebral regional oxygen saturation were also significantly higher in the 80% oxygen group.

Previous studies on the effects of supplemental oxygen have been largely performed in critically ill patients [22, 23]. Although some landmark trials in patients undergoing surgery have been published [13, 14], in contrast to that in critically ill patients, the evidence on the topic is relatively insufficient and does not reflect different surgical settings [24]. Moreover, most prior studies in cardiac surgical patients enrolled only a small number of patients with cardiopulmonary bypass and had markedly heterogeneous study designs (e.g., varying FiO_2_ or PaO_2_ targets used) and primary outcomes [11]. The most recent randomized trial was performed in 330 patients undergoing cardiopulmonary bypass and failed to find any difference in clinical outcomes between the standard management (varying FiO_2_ to achieve PaO_2_ < 150 mmHg during cardiopulmonary bypass) and the intervention (FiO_2_ of 1.0) [25]. Consequently, there is no consensus regarding adequate oxygen therapy in cardiac surgical patients, particularly in those undergoing off-pump cardiac surgery, and thus real-world clinical practice has varied [26]. One of the main reasons for this complexity is that clinical advantages and disadvantages of a specific oxygen level can vary from organ to organ. The present CARROT trial is the first randomized clinical trial performed in patients undergoing off-pump cardiac surgery on this topic. Although no difference was found in the primary clinical endpoint, we showed that the use of 80% oxygen compared to 30% oxygen reduced postoperative AKI and improved hemodynamics without any disadvantage.

Although some cohort studies showed that increased oxygen exposure was associated with a higher risk of acute kidney injury after major surgery [27], there has been no conclusive evidence that supplemental oxygen alters renal outcome after cardiac surgery [28]. In our study, although exploratory in nature, postoperative AKI was considerably reduced in the 80% oxygen group compared to the 30% oxygen group, and this result was consistent with a lower mean serum NGAL level at the end of surgery in the 80% oxygen group (91 ng/ml vs 133 ng/ml). Furthermore, well-established risk factors for AKI, such as hemoglobin concentration (anemia) and arterial blood pressure [29, 30], were nearly identical between the two groups (see Table 4). We infer an increased DO_2_ as a possible mechanism underlying the beneficial effect of a higher FiO_2_ on postoperative AKI. In our previous substudy of this CARROT trial, we already showed that a significantly higher DO_2_ can be achieved by using 80% oxygen compared with 30% oxygen in OPCAB patients [31]. Likewise, in the present study, SvO_2_, an index of the balance between DO_2_ and oxygen consumption, was also higher in the 80% oxygen group, while CO and hemoglobin concentration were similar between the two groups (see Table 5). The depth of anesthetics may be an important factor that influences critical DO_2_ and changes the intra-organ pressure-flow relationship [32]. In our study, however, the mean time-weighted average bispectral index was nearly identical among the two groups (42.3 [4.6] and 43.2 [4.5] in the 30% and 80% oxygen groups, respectively; data not shown in the Results). Then, could the DO_2_ difference observed in this study reach a threshold where the incidence of postoperative AKI increases? Peritubular capillaries of the kidney are supplied by efferent glomerular arteries that carry poorly oxygenated blood [33], which is particularly prominent in the renal medulla [34]. Indeed, oxygen tension in the renal medulla is among the lowest in the body [35, 36]. Thus, it is not surprising that the kidney is vulnerable to the reduction in DO_2_, and conversely, increasing DO_2_ may ameliorate renal ischemic injury [37]. In our exploratory calculation, the mean (standard deviation) intraoperative time-weighted average indexed DO_2_ was 305 (63) and 289 (59) ml/min/m^2^ in the 80% and 30% oxygen groups, respectively. Considering that a cut-off for indexed DO_2_ value known to predict AKI was at approximately 270 ml/min/m^2^ [38, 39], more patients in the 30% oxygen group might have failed to achieve this cut-off value. As expected, in the present study, patients who developed AKI showed a lower indexed DO_2_ than those who did not (279 [52] ml/min/m^2^ vs 303 [60] ml/min/m^2^). The authors looked further into the data, stratifying patients by whether intraoperative time-weighted average indexed DO_2_ was lower than 270 ml/min/m^2^ (see Additional file 1: Figure S4). Interestingly, there was little difference in the incidence of postoperative AKI between the 30% and 80% oxygen groups (16.5% vs 16.8%) within the ‘high DO_2_’ stratum. The difference in time-weighted average CO was trivial (4.0 l/min vs 3.9 l/min). On the other hand, within the ‘low DO_2_’ stratum, the incidence of AKI in the 30% and 80% oxygen groups was 34.6% and 20.9%, respectively, while the time-weighted average CO was 3.1 l/min and 3.0 l/min. First, the difference in DO_2_ between the DO_2_ strata appeared to be mainly due to the difference in CO. We infer that patients with relatively high CO did not benefit from 80% oxygen because DO_2_ was already sufficient, whereas those with lower CO did. Taken together, it may be helpful to raise DO_2_ by increasing FiO_2_ in patients with low CO for the prevention of postoperative AKI. Based on these findings and hypothesis, the authors expect that the effects of a high FiO_2_ may be advantageous in specific hemodynamic environments such as OPCAB where low CO and hypotension are frequent [12], although it may not be applicable in other various surgical settings.

In general, lung injury from a high FiO_2_ has been acknowledged in critically ill patients [17, 40]. A prior randomized controlled trial demonstrated that targeting a higher PaO_2_ significantly increased mortality in critically ill patients [17]. However, a recent study found no difference in ventilator-free days between patients where a lower, intermediate, or higher oxygen saturation target was used [41]. Similarly, there have been inconsistent results in surgical patients [42]. In a previous large observational study, high intraoperative FiO_2_ was associated with a composite of postoperative respiratory complications (reintubation, respiratory failure, pulmonary edema, and pneumonia) in a dose-dependent manner [43]. However, a meta-analysis showed a nonsignificant association of a high intraoperative FiO_2_ with respiratory insufficiency after surgery [42]. In the SO-COOL trial by McGuinness et al., MV time after cardiac surgery involving cardiopulmonary bypass was comparable between the usual care and the avoidance of hyperoxemia groups [44]. In our study, we found no significant difference in MV time, incidence of prolonged MV, and initial PaO_2_/FiO_2_ ratio after OPCAB between the 30% and 80% oxygen groups. Interestingly, MV time was shorter and the incidence of prolonged MV was lower in the 80% oxygen group (see Fig. 3).

Since the World Health Organization recommended using 80% oxygen to reduce surgical site infection, the effect of perioperative FiO_2_ on surgical site infection has been one of the most contentious issues [45]. Despite debates and criticism [46–48], the World Health Organization still recommends using 80% oxygen for the prevention of surgical site infection in the updated guideline [16]. However, a recent large trial and a meta-analysis reported no or little effect of hyperoxia on surgical site infection [49, 50], and so far, evidence supporting the use of 80% oxygen to prevent surgical site infection was largely from colorectal surgery cohorts. In our study, sternal wound infection was less frequent in the 80% oxygen group than in the 30% oxygen group (see Fig. 3), although it was statistically nonsignificant. However, conclusive interpretation was not possible due to a low event rate and a different study population in our study.

There have been concerns about hyperoxia-related myocardial injury after noncardiac surgery (MINS) [51], and even myocardial infarction [52]. In the present study, there was no significant difference between the 80% and 30% oxygen groups in terms of postoperative serum troponin I and creatine kinase MB concentrations and type 5 myocardial infarction, while serum troponin T concentration measured in one participating center was rather lower in the 80% oxygen group than in the 30% oxygen group (see Table 3). In fact, previous studies also showed inconsistent results about the relationship between perioperative FiO_2_ and myocardial injury [28, 51, 53]. Two recent prospective controlled trials reported a nonsignificant difference in the risk for MINS between 80 and 30% oxygen [28, 53]. On the contrary, the most recent international observational study showed that an intraoperative FiO_2_ increment of 0.1 was associated with an increase in odds for MINS (odds ratio, 1.17; 95% CI 1.12–1.23) [51]. Further studies are required to conclude this issue, but in our study, evidence of increased myocardial injury was not observed in the 80% oxygen group compared to 30% oxygen group.

We recognize several limitations of this study. First, intraoperative FiO_2_ levels were randomized in this study instead of specific PaO_2_ targets. Even at the same FiO_2_, arterial blood oxygenation may vary substantially depending on the individual lung condition. Although PaO_2_ was not measured at prespecified time points during surgery, it is very likely that treatment separation was well achieved considering the mean difference in time-weighted average PaO_2_ among the two groups (124 mmHg vs 316 mmHg; see Table 4). Moreover, we expected that interventions based on FiO_2_ rather than PaO_2_ targets could be more pragmatic during clinical practice. Second, although the primary endpoint of this study was the hospital LOS, criteria for hospital discharge were not protocolized. This may have contributed to the failure to detect the difference in hospital LOS between the study groups. However, we expected that this aspect made the present study pragmatic and better reflect the real-world clinical situation. Third, the exploratory secondary clinical outcomes including postoperative AKI were not prespecified in a clinical trial registry, thereby having an exploratory nature despite the significant difference in the incidence of AKI. Fourth, we regulated intraoperative FiO_2_ level in the study protocol, but not postoperative supplemental oxygen therapy. Considering the importance of postoperative management, the benefits from high FiO_2_ might have been more evident, or even an opposite conclusion could have been reached, had the postoperative FiO_2_ level been also regulated. Further studies are warranted for this topic.

In conclusion, intraoperative administration of 80% oxygen or 30% oxygen did not affect hospital LOS after OPCAB. However, 80% oxygen during OPCAB may decrease the incidence of postoperative AKI and serum NGAL concentration. Furthermore, global oxygen supply and cerebral regional oxygen saturation were also superior in the 80% oxygen group to the 30% oxygen group. Although it failed to reduce hospital LOS, an intraoperative FiO_2_ of 0.8 provided tissue oxygenation with superior hemodynamics without any worse outcome related to hyperoxia.

## Supplementary Information

**Additional file 1.** Supplementary materials for the CARROT study.

## Data Availability

The datasets used and/or analyzed during the current study are available from the corresponding authors on reasonable request.
