# Supplementary material for: Effects of intraoperative inspired oxygen fraction (FiO2 0.3 vs 0.8) on patients undergoing off-pump coronary artery bypass grafting: the CARROT multicenter, cluster-randomized trial
Source: Crit Care. 2023 Jul 13;27:286. doi: 10.1186/s13054-023-04558-8 (PMC10339585; doi:10.1186/s13054-023-04558-8)
Supplement: Supplementary file 1 — Additional file 1. Supplementary materials for the CARROT study. [file 13054_2023_4558_MOESM1_ESM.docx]

**Supplementary Material**

**Effects of intraoperative inspired oxygen fraction (FiO_2_ 0.3 vs 0.8) on patients undergoing off-pump coronary artery bypass grafting: the CARROT multicenter, cluster-randomized trial**

Karam Nam,^1,a^ Jae-Sik Nam,^2,a^ Hye-Bin Kim,^3,b^ Jaeyeon Chung,^1,c^ In Eob Hwang,^1^ Jae-Woo Ju,^1^ Jinyoung Bae,^1,d^ Seohee Lee,^1^ Youn Joung Cho,^1^ Jae‑Kwang Shim,^3^ Young-Lan Kwak,^3^ Ji-Hyun Chin,^2^ In-Cheol Choi,^2^ Eun-Ho Lee,^2,e,f^ Yunseok Jeon^1,f^ for the CARdiac suRgery and Oxygen Therapy (CARROT) Investigators

^1^Department of Anesthesiology and Pain Medicine, Seoul National University Hospital, Seoul National University College of Medicine, Seoul, Republic of Korea

^2^Department of Anesthesiology and Pain Medicine, Asan Medical Center, University of Ulsan College of Medicine, Seoul, Republic of Korea

^3^Department of Anesthesiology and Pain Medicine, Severance Hospital, Anesthesia and Pain Research Institute, Yonsei University College of Medicine, Seoul, Republic of Korea

^b^Current affiliation: Department of Anesthesiology and Pain Medicine, Korea University Guro Hospital, Korea University College of Medicine, Seoul, Republic of Korea

^c^Current affiliation: Medical Service Corps of the First Logistics Support Command, Wonju, Gangwon State, Republic of Korea

^d^Current affiliation: Department of Anesthesiology and Pain Medicine, Ajou University Medical Center, Ajou University School of Medicine, Suwon, Gyeonggi Province, Republic of Korea

^e^Current affiliation: Hana Anesthesia Clinic, Seoul, Republic of Korea

^a^K.N. and J.-S.N. contributed equally to this work and share the role of first author.

^f^E.-H.L. and Y.J. contributed equally to this work and share the role of corresponding author.

**Correspondence to:** Eun-Ho Lee (leho@naver.com) and Yunseok Jeon (jeonyunseok@gmail.com)

*E.-H.L.*

Department of Anesthesiology and Pain Medicine

Asan Medical Center, University of Ulsan College of Medicine

88 Olympic-ro 43-gil, Songpa-gu, Seoul 05505, Republic of Korea

Email: leho@naver.com

Tel.: +82 10 2315 0768

*Y.J.*

Department of Anesthesiology and Pain Medicine

Seoul National University Hospital, Seoul National University College of Medicine

101 Daehak-ro, Jongno-gu, Seoul 03080, Republic of Korea

Tel.: +82 2 2072 3108

**TABLE OF CONTENTS**

**Supplementary Methods**… p.4

Table S1. Summary of the routine perioperative management in each participating hospital… p.4

CARROT Clinical outcome event definitions… p.7

Table S2. Definition of a superficial sternal wound site infection used for the study… p.9

Table S3. Definition of a deep sternal wound infection used for the study… p.10

Table S4. Definition of mediastinitis used for the study… p.11

**Supplementary Results**… p.12

Figure S1. The distribution of sample size according to clusters and participating hospitals… p.12

Table S5. Time-weighted average values of intraoperative mechanical ventilatory parameters of patients in one participating hospital (SNUH)… p.13

Table S6. Unplanned readmission… p.14

Table S7. Per-protocol analysis of the continuous secondary clinical outcomes… p.15

Figure S2. Per-protocol analysis of the binary secondary clinical outcomes… p.16

Table S8. Per-protocol analyses of biochemical outcomes… p.17

Figure S3. Box plots of first postoperative serum lactate concentration according to the study groups… p.18

Table S9. Per-protocol analyses of hemodynamic data… p.19

Figure S4. Post-hoc analysis of the incidence of AKI according to intraoperative TWA indexed DO_2_ and FiO_2_… p.20

**References for the Supplementary Materials**… p.21

**Supplementary Methods**

**Table S1.** Summary of the routine perioperative managements in each participating hospital

|  | **Seoul National University Hospital** | **Asan Medical Center** | **Severance Hospital** |
| --- | --- | --- | --- |
| **Preoperative antibiotic prophylaxis** | Cefuroxime 750 mg before skin incision. The same dose repeated every 8 h until POD 1. | Cefazolin 2 g before skin incision.  The same dose repeated 4 h after skin incision, and then 1 g after 8 h. | Flomoxef 1 g before skin incision.  The same dose repeated every 12 h until POD 2. |
| **Intraoperative mechanical ventilation** | - Tidal volume: 6–8 ml/kg (IBW) - PEEP: 0 cmH_2_O - Respiratory rate: to maintain EtCO_2_ between 30–40 mmHg | - Tidal volume: 6–8 ml/kg (IBW) - PEEP: 5 cmH_2_O initially, and then reduced to 0–3 cmH_2_O after sternotomy - Respiratory rate: to maintain EtCO_2_ between 30–40 mmHg | - Tidal volume: 6–8 ml/kg (IBW) - PEEP: 0 cmH_2_O - Respiratory rate: to maintain EtCO_2_ between 30–40 mmHg |
| **Intraoperative ABGA** | - Interval: every 1–1.5 h after anesthesia induction. Additional measurements at the attending anesthetist’s discretion. - Device: GemPremier3000 (Instrumentation Laboratory, Bedford, MA, USA) | - Timing: i) after induction, ii) after heparin administration, iii) after left anterior descending artery anastomosis, and iv) after all anastomoses are finished. Additional measurements at the attending anesthetist’s discretion. - Device: GemPremier3500 (Instrumentation Laboratory, Bedford, MA, USA) | - Timing: i) baseline, ii) 5 min after heparin administration, iii) 15 min after protamine administration, and iv) 5 min after sternal closure. Additional measurements at the attending anesthetist’s discretion. - Device: Stat Profile pHOx Ultra Blood Gas Analyzer (Nova Biomedical, Waltham, MA, USA) |
| **Transfusion trigger** | Hemoglobin: <8–9 g/dl | Hemoglobin: <8 g/dl | Hemoglobin: <7–8 g/dl |
| **First-line intraoperative vasopressors and inotropes** | - Rescue bolus: phenylephrine or ephedrine - Continuous: norepinephrine | - Rescue bolus: phenylephrine, ephedrine, or norepinephrine - Continuous: norepinephrine, dobutamine | - Rescue bolus: ephedrine or vasopressin - Continuous: norepinephrine, milrinone |
| **Postoperative mechanical ventilation** | - Initial FiO_2_: 0.6–0.8 - Extubation criteria: oxygen saturation >94% and PaO_2_ >80 mmHg on an FiO_2_ <0.5 and PEEP <8 cmH_2_O (the final decision was at the attending intensivist’s discretion) | - Initial FiO_2_: 0.6 - Extubation criteria: oxygen saturation >95%, PaO_2_ >70 mmHg, and pH ≥7.30 on an FiO_2_ ≤0.4, PEEP ≤5 cmH_2_O, pressure support level ≤10 cmH_2_O, and respiratory rate <30 breaths/min (the final decision was at the attending intensivist’s discretion) | - Initial FiO_2_: 0.4–0.5 - Extubation criteria: oxygen saturation >94% and PaO_2_ >80 mmHg on an FiO_2_ <0.5 and PEEP <8 cmH_2_O (the final decision was at the attending intensivist’s discretion) |
| **Postoperative sedatives** | Dexmedetomidine ± remifentanil | Dexmedetomidine ± remifentanil | Propofol ± remifentanil |
| **Postoperative serum creatinine measurement** | During ICU stay: at 6 h after surgery, and every 8 h from POD 1 until ICU discharge.  During ward stay: daily until POD 4–7.  Additional measurements at the attending physician’s discretion. | Daily until POD 3.  Additional measurements at the attending physician’s discretion. | Daily until POD 7.  Additional measurements at the attending physician’s discretion. |
| **Postoperative high-sensitivity troponin and CK-MB measurement** | During ICU stay: at 6 h after surgery, and then daily during ICU stay.  During ward stay: daily until the attending physician decided to stop measuring at his/her discretion. | Immediate postop and POD 1.  Additional measurements at the attending physician’s discretion. | Immediate postop, POD 1, 2, and 7.  Additional measurements at the attending physician’s discretion. |
| **Postoperative serum lactate measurement** | At the attending intensivist’s discretion during ICU stay. | At the attending intensivist’s discretion during ICU stay. | Immediate postop, and daily during ICU stay. |

POD, postoperative day; IBW, ideal body weight; PEEP, positive end expiratory pressure; EtCO_2_, end-tidal carbon dioxide partial pressure; ABGA, arterial blood gas analysis; FiO2, fraction of inspired oxygen; PaO2, arterial oxygen partial pressure; ICU, intensive care unit; CK-MB; creatine kinase MB.

**CARROT Clinical outcome event definitions**

The secondary postoperative clinical outcomes listed below were events reported during the index hospitalization for surgery, if not otherwise specified.

- **Prolonged mechanical ventilation:** mechanical ventilation for more than 48 hours.
- **In-hospital mortality:** death from any cause before discharge regardless of timing.
- **Delirium** diagnosed by board-certified, consultation-liaison psychiatrists.
- **Stroke:** a new ischemic or hemorrhagic cerebrovascular accident with a neurological deficit confirmed by brain imaging.
- **Sternal wound infection:** superficial or deep sternal wound infection or mediastinitis after off-pump coronary artery bypass, defined using the National Healthcare Safety Network surgical site infection surveillance definition of the Centers for Disease Control and Prevention. See Tables S2, S3, and S4 below (pp. 9–11).
- **Acute kidney injury** developed within 7 days after surgery, defined based on the serum creatinine criteria of the Kidney Disease: Improving Global Outcomes (KDIGO).^1^
  - Increase in serum creatinine level by 0.3 mg/dl or more within 48 hours, or
  - Increase in serum creatinine level to 1.5 times the baseline or more within 7 days.
  - The baseline serum creatinine level was defined as the most recent value measured prior to surgery.
- **New-onset atrial fibrillation** of any duration captured on 12-lead electrocardiogram, continuous electrocardiogram monitoring, or telemetry.
- **Type 5 myocardial infarction ≤48 hours after surgery** diagnosed based on the Fourth Universal Definition of Myocardial Infarction.^2^
  - Increase of cardiac troponin >10 times the 99th percentile upper reference limit in patients with normal baseline values.
  - In patients with elevated preoperative cardiac troponin (in whom cardiac troponin levels are stable or falling), postoperative cardiac troponin level must increase by >20%. However, the absolute postoperative cardiac troponin value still must be >10 times the 99th percentile upper reference limit.
  - In addition, one of the followings is required:
    - Development of new pathological Q waves.
    - Angiographically documented flow-limiting complication such as coronary artery dissection, occlusion of a native coronary artery or graft, side-branch occlusion-thrombus, disruption of collateral flow, or distal embolization.
    - Imaging evidence of new loss of viable myocardium or regional wall motion abnormality in a pattern consistent with an ischemic etiology.
  - Isolated development of new pathological Q waves meets the type 5 myocardial infarction criteria if cardiac troponin values are elevated and rising but <10 times the 99th percentile upper reference limit.
- **Revascularization:** percutaneous coronary intervention or bypass grafting within 30 days after the initial coronary artery bypass grafting, which was not an a priori planned stepwise procedure.

**Table S2.** Definition of *superficial* sternal wound site infection used for the study

| *Superficial* sternal wound infection must meet the following criteria:  Date of event occurs within 30 days after OPCAB  *AND*  involves only skin and subcutaneous tissue of the incision  *AND*  patient has at least one of the followings:  a. purulent discharge from the superficial incision  b. organism(s) identified from an aseptically-obtained specimen from the superficial incision or subcutaneous tissue by a culture-based microbiologic testing method which is performed for purposes of clinical diagnosis or treatment.  c. superficial incision that is deliberately opened by a physician and culture-based testing of the superficial incision or subcutaneous tissue is not performed.  *AND*  patient has at least one of the following signs or symptoms: localized pain, tenderness, swelling, erythema, or heat.  d. diagnosis of a superficial incisional surgical site infection by a physician. |
| --- |

Adapted from the *Patient Safety Component Manual [Chapter 9: Surgical Site Infection Event]* of the *National Healthcare Safety Network* of the *Centers for Disease Control and Prevention*.

OPCAB, off-pump coronary artery bypass grafting.

**Table S3.** Definition of a *deep* sternal wound infection used for the study

| *Deep* sternal wound infection must meet the following criteria:  Date of event occurs within 90 days after OPCAB  *AND*  involves deep soft tissues of the incision (for example, fascial and muscle layers)  *AND*  patient has at least one of the following:  a. purulent drainage from the deep incision.  b. a deep incision that spontaneously dehisces, or is deliberately opened or aspirated by a physician  *AND*  organism(s) identified from the deep soft tissues of the incision by a culture-based microbiologic testing method which is performed for purposes of clinical diagnosis or treatment or culture-based microbiologic testing method is not performed. A culture-based test from the deep soft tissues of the incision that has a negative finding does not meet this criterion.  *AND*  patient has at least one of the following signs or symptoms: fever (>38°C), localized pain or tenderness.  c. an abscess or other evidence of infection involving the deep incision that is detected on gross anatomical or histopathologic exam, or imaging test. |
| --- |

Adapted from the *Patient Safety Component Manual [Chapter 9: Surgical Site Infection Event]* of the *National Healthcare Safety Network* of the *Centers for Disease Control and Prevention*.

OPCAB, off-pump coronary artery bypass grafting.

**Table S4.** Definition of *mediastinitis* used for the study

| Mediastinitis must meet the following criteria:  Date of event occurs within 90 days after OPCAB  *AND*  patient has at least one of the following:  a. patient has organism(s) identified from mediastinal tissue or fluid by a culture-based microbiologic testing method which is performed for purposes of clinical diagnosis or treatment.  b. patient has evidence of mediastinitis on gross anatomic or histopathologic exam.  c. patient has at least one of the following signs or symptoms: fever (>38.0°C), chest pain, or sternal instability.  *AND* *at least one of the following:*  i) purulent drainage from mediastinal area  ii) mediastinal widening on imaging test |
| --- |

Adapted from the *Patient Safety Component Manual [Chapter 17: CDC/NHSN Surveillance Definitions for Specific Types of Infections]* of the *National Healthcare Safety Network* of the *Centers for Disease Control and Prevention*.

OPCAB, off-pump coronary artery bypass grafting.

**Supplementary Results**

**Figure S1.** The distribution of sample size according to clusters and participating hospitals**
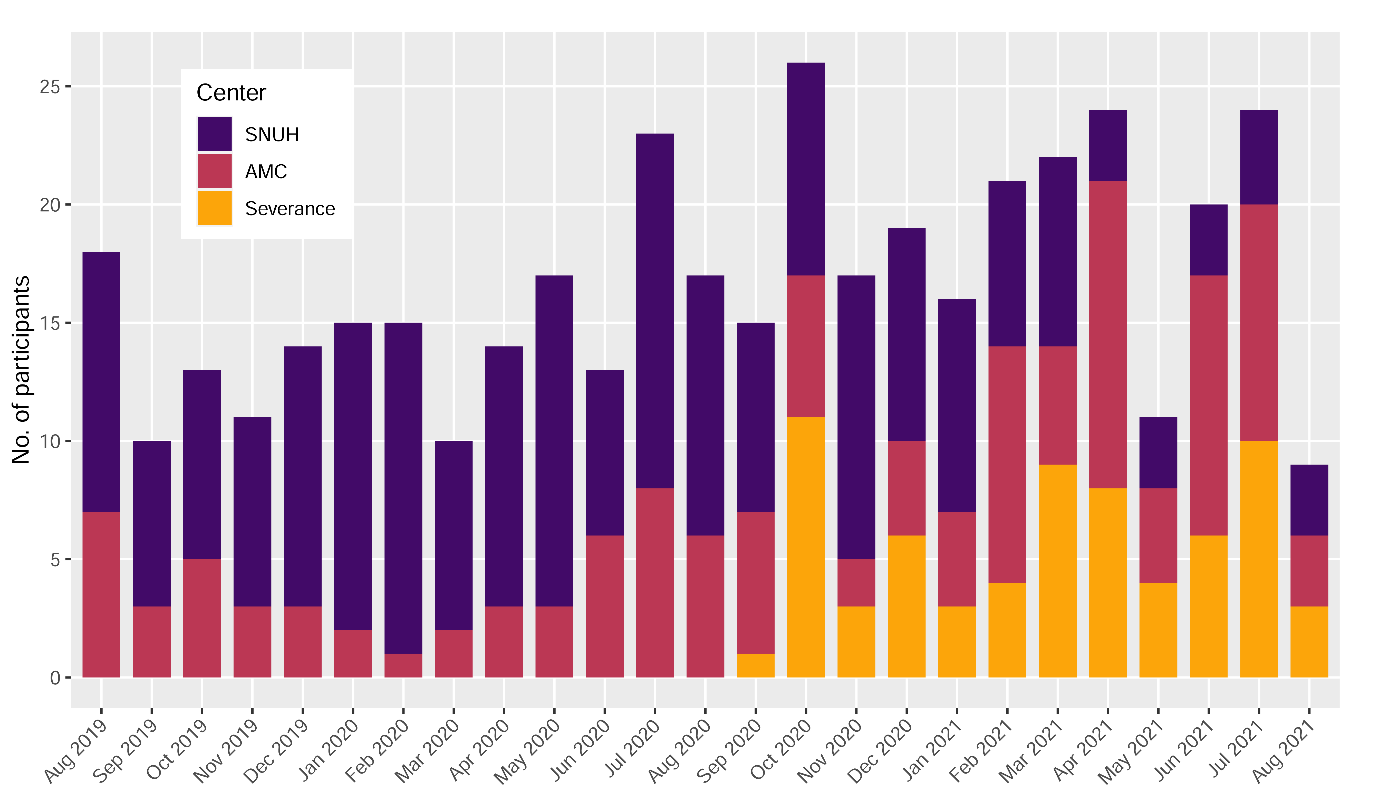
**

**Table S5.** Time-weighted average values of intraoperative mechanical ventilatory parameters of patients in one participating hospital (SNUH)

| Ventilatory parameters | 30% oxygen  (n = 113) | 80% oxygen  (n = 103) |
| --- | --- | --- |
| Peak inspiratory pressure (cmH_2_O) | 14 (2) | 14 (3) |
| PEEP (cmH_2_O) | 2 (1) | 2 (1) |
| V_T_/IBW (ml/kg) | 7.4 (0.9) | 7.6 (1.1) |
| Respiratory rate (breaths per minute) | 13 (2) | 13 (2) |
| Minute ventilation (l) | 5.6 (1.0) | 5.6 (1.2) |

Data are presented as mean (standard deviation). SNUH, Seoul National University Hospital; PEEP, positive end-expiratory pressure; V_T_, tidal volume; IBW, ideal body weight.

**Table S6.** Unplanned readmission

| Intention-to-treat analysis^a^ | | 30% oxygen  (n = 113) | | 80% oxygen  (n = 103) | | Odds ratio^b^ or estimate^c^ (95% CI)^d^ | | *P* | |
| --- | --- | --- | --- | --- | --- | --- | --- | --- | --- |
| Readmission within 60 days after discharge from index hospitalization | | 7 (6.2%) | | 6 (5.8%) | | 1.07 (0.42–2.74) | | 0.892 | |
| No. of days in hospital after readmission | | 1.6 (7.0) | | 1.6 (13.2) | | 0 (-2.8–2.7) | | 0.958 | |
| Causes of readmission | |  | |  | |  | |  | |
| Sternal wound infection | | 4 (3.5%) | | 3 (2.9%) | |  | |  | |
| Pleural or pericardial effusion | | 2 (1.8%) | | 1 (1.0%) | |  | |  | |
| Miscellaneous | | 1 (0.9%) | | 2 (1.9%) | |  | |  | |
| Per-protocol analysis^a^ | | 30% oxygen  (n = 111) | | 80% oxygen  (n = 100) | | Odds ratio^b^ or estimate^c^ (95% CI)^d^ | | *P* | |
| Readmission within 60 days after discharge from index hospitalization | | 7 (6.3%) | | 5 (5.0%) | | 1.28 (0.45–3.62) | | 0.643 | |
| No. of days in hospital after readmission | | 1.6 (7.1) | | 1.6 (13.4) | | 0 (-2.8–2.8) | | 0.986 | |
| Causes of readmission | |  | |  | |  | |  | |
| Sternal wound infection | | 4 (3.6%) | | 3 (3.0%) | |  | |  | |
| Pleural or pericardial effusion | | 2 (1.8%) | | 0 (0%) | |  | |  | |
| Miscellaneous | | 1 (0.9%) | | 2 (2.0%) | |  | |  | |

Data were presented as number (proportion) or mean (standard deviation).

CI, confidence interval.

^a^Results from one participating hospital (Seoul National University Hospital).

^b^For a binary outcome.

^c^For a continuous outcome.

^d^Referenced to the 80% oxygen group.

**Table S7.** Per-protocol analysis of the continuous secondary clinical outcomes

|  | 30% oxygen  (n = 203) | 80% oxygen  (n = 202) | Estimate (95% CI)^a^ | *P*^b^ |
| --- | --- | --- | --- | --- |
| Initial postoperative PaO_2_/FiO_2_ ratio^c^ | 316 (109) | 289 (110) | 26 (-1–53) | 0.156 |
| ICU length of stay, hours | 50.0 (41.9) | 52.0 (41.7) | -2.1 (-9.5–5.4) | 0.822 |
| MV time, hours | 14.8 (18.4) | 12.1 (7.2) | 3.1 (-0.3–6.6) | 0.170 |

Data are presented as mean (standard deviation) or number (proportion). Definitions of the outcomes are provided in page 3 above.

CI, confidence interval; PaO_2_, arterial oxygen partial pressure; FiO_2_, inspired oxygen fraction; ICU, intensive care unit; MV, mechanical ventilation.

^a^Referenced to the 80% oxygen group.

^b^False discovery rate-corrected values.

^c^The results are from one participating hospital (n = 111 and 100 in the 30% and 80% oxygen groups, respectively).

**Figure S2.** Per-protocol analysis of the binary secondary clinical outcomes

**
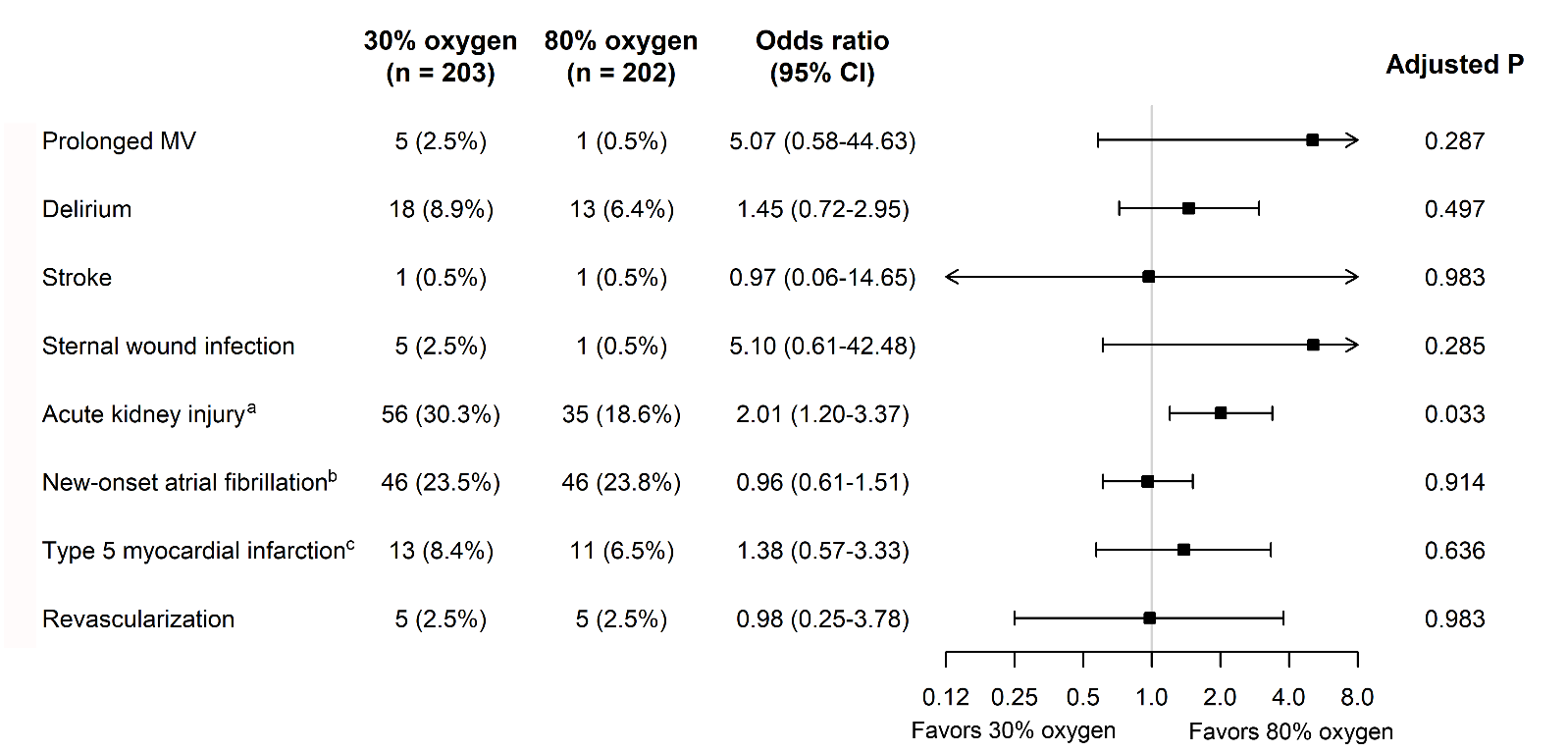
**

CI, confidence interval; MV, mechanical ventilation.

^a^18 and 14 patients with preoperative end-stage renal disease or renal replacement therapy in the 30% and 80% oxygen groups, respectively, were excluded.

^b^7 and 9 patients with a preoperative history of atrial fibrillation in the 30% and 80% oxygen groups, respectively, were excluded.

^c^Missing data in 49 and 34 patients in the 30% and 80% oxygen groups, respectively.

**Table S8.** Per-protocol analysis of biochemical outcomes

|  | 30% oxygen  (n = 203) | 80% oxygen  (n = 202) | Estimate (95% CI)^a^ | *P^b^* |
| --- | --- | --- | --- | --- |
| 72 h maximum cTnT, pg/ml^c^ | 0.52 (0.88) | 0.23 (0.22) | 0.29 (0.06–0.51) | 0.040 |
| 72 h AUC cTnT, pg/ml·h^c^ | 26.2 (44.1) | 10.3 (8.6) | 15.7 (4.6–26.7) | 0.025 |
| 72 h maximum cTnI, ng/ml^d^ | 2.45 (4.96) | 2.87 (5.52) | -0.42 (-1.52–0.68) | 0.636 |
| 72 h AUC cTnI, ng/ml·h^d^ | 57.2 (125.8) | 69.8 (154.9) | -11.3 (-37.3–14.6) | 0.578 |
| 72 h maximum CKMB, ng/ml | 9.30 (18.73) | 8.69 (9.12) | 0.73 (-2.21–3.66) | 0.766 |
| 72 h AUC CKMB, ng/ml·h | 249.2 (487.6) | 243.5 (278.4) | 16.9 (-60.7–94.5) | 0.781 |
| First postoperative lactate, mmol/l | 1.1 (0.5) | 1.0 (0.4) | 0.06 (-0.03–0.15) | 0.353 |
| NGAL, at the end of surgery, ng/ml^e^ | 132.0 (206.1) | 84.0 (77.9) | 47.1 (9.3–84.9) | 0.046 |

Data are presented as mean (standard deviation). CI, confidence interval; cTnT, cardiac troponin T; AUC, area under the curve; cTnI, cardiac troponin I; CKMB, creatine kinase MB; NGAL, neutrophil gelatinase-associated lipocalin.

^a^Referenced to the 80% oxygen group.

^b^False discovery rate-corrected values.

^c^n = 25 and 43 in the 30% and 80% oxygen groups, respectively.

^d^n = 178 and 159 in the 30% and 80% oxygen groups, respectively.

^e^n = 100 and 93 in the 30% and 80% oxygen groups, respectively.

**Figure S3.** Box plots of first postoperative serum lactate concentration according to the study groups

**
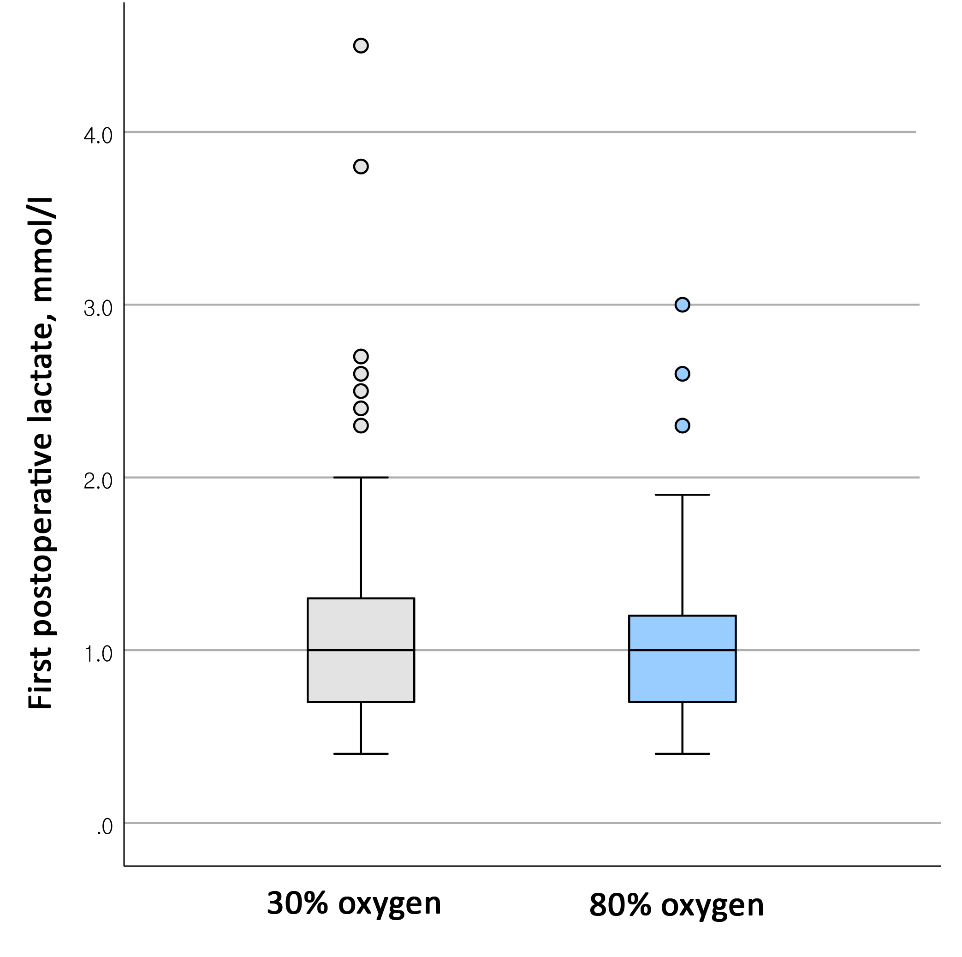
**

**Table S9.** Per-protocol analysis of hemodynamic data

|  | 30% oxygen  (n = 203) | 80% oxygen  (n = 202) | Estimate (95% CI)^a^ | *P^b^* |
| --- | --- | --- | --- | --- |
| Hemoglobin, g/dl^a^ | 10.2 (1.2) | 10.5 (1.4) | -0.2 (-0.5–0.0) | 0.144 |
| SaO_2_, %^a^ | 97.9 (1.5) | 99.8 (0.4) | -1.9 (-2.1–-1.6) | 0.001 |
| PaO_2_, mmHg^a^ | 123 (29) | 317 (62) | -198 (-210–-186) | 0.001 |
| SvO_2_, %^a^ | 64.0 (9.7) | 73.9 (7.4) | -8.7 (-10.3–-7.1) | 0.001 |
| Cardiac output, l/min^b^ | 3.7 (0.7) | 3.6 (0.8) | 0.1 (-0.1–0.3) | 0.567 |
| Cardiac index, l/min/m^2b^ | 2.1 (0.4) | 2.1 (0.4) | 0.1 (0.0–0.2) | 0.351 |
| Cerebral rSO_2_, %^c^ | 51.9 (14.2) | 56.7 (15.8) | -5.0 (-6.6–-3.4) | 0.001 |
| MBP, mmHg | 74 (6) | 76 (6) | -1.5 (-2.5–-0.6) | 0.012 |

All values are time-weighted average intraoperative values. Data are presented as mean (standard deviation).

CI, confidence interval; SaO_2_, arterial oxygen saturation; PaO_2_, arterial oxygen partial pressure; SvO_2_, mixed venous oxygen saturation; rSO_2_, regional oxygen saturation; MBP, mean blood pressure.

^a^Referenced to the 80% oxygen group.

^b^False discovery rate-corrected values.

^c^n = 147 and 145 in the 30% and 80% oxygen groups, respectively.

^d^The lower of the left and right side values was taken.

**Figure S4**. Post-hoc analysis of the incidence of postoperative AKI according to intraoperative TWA indexed DO_2_ and FiO_2_


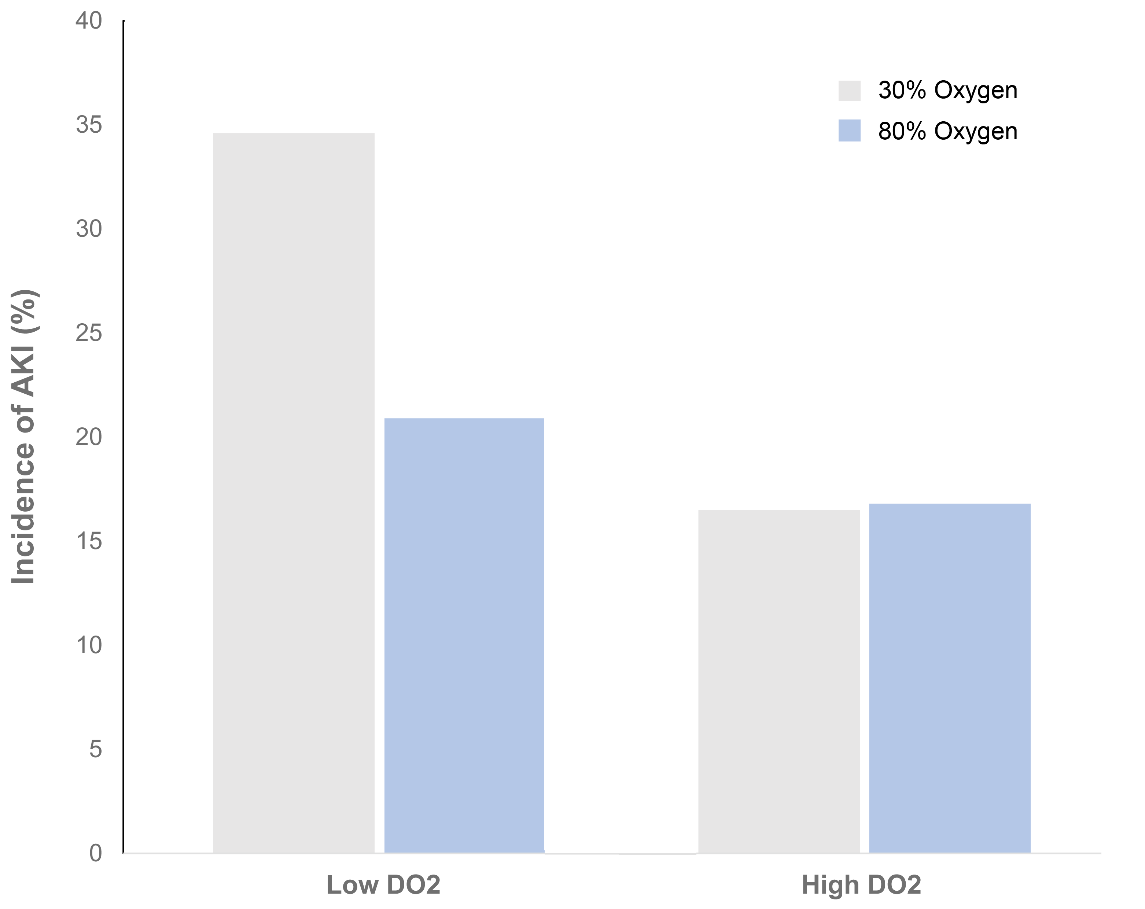


Analysis of 297 patients with no missing data. Low and high DO_2_ refer to intraoperative TWA indexed DO_2_ of ≤ and >270 ml/min/m^2^, respectively.

AKI, acute kidney injury; TWA, time-weighted average; DO_2_, oxygen delivery; FiO_2_, fraction of inspired oxygen.

**References for the Supplementary Materials**

1. Kidney Disease: Improving Global Outcomes (KDIGO) Acute Kidney Injury Work Group. KDIGO Clinical Practice Guideline for acute kidney injury. Kidney Int Suppl 2012;2:1-138.

2. hygesen K, Alpert JS, Jaffe AS, Chaitman BR, Bax JJ, Morrow DA, et al. Fourth Universal Definition of Myocardial Infarction (2018). J Am Coll Cardiol 2018;72:2231-2264.
